# The TIR-domain-containing adapter inducing interferon-β-dependent signaling cascade plays a crucial role in ischemia–reperfusion-induced retinal injury, whereas the contribution of the myeloid differentiation primary response 88-dependent signaling cascade is not as pivotal

**DOI:** 10.1111/ejn.12603

**Published:** 2014-04-23

**Authors:** Galina Dvoriantchikova, Andrea Rachelle C Santos, Dagmara Danek, Xenia Dvoriantchikova, Dmitry Ivanov

**Affiliations:** 1 Department of Ophthalmology, Bascom Palmer Eye Institute, University of Miami Miller School of Medicine1638 NW 10th Ave, Miami, FL, 33136, USA; 2 Department of Microbiology and Immunology, University of Miami Miller School of MedicineMiami, FL, USA

**Keywords:** inflammation, ischemia–reperfusion injury, mouse models, retinal damage, toll-like receptor signaling

## Abstract

Toll-like receptor 4 (Tlr4) plays an important role in ischemia–reperfusion (IR)-induced retinal inflammation and damage. However, the role of two Tlr4-dependent signaling cascades, myeloid differentiation primary response 88 (Myd88) and TIR-domain-containing adapter inducing interferon-β (Trif), in retinal IR injury is poorly understood. In this study, we investigated the contribution of the Myd88-dependent and Trif-dependent signaling cascades in retinal damage and inflammation triggered by IR, by using *Myd88* knockout (*Myd88*KO) and *Trif* knockout (*Trif*KO) mice. Retinal IR injury was induced by unilateral elevation of intraocular pressure for 45 min by direct corneal cannulation. To study IR-induced retinal ganglion cell (RGC) death *in vitro*, we used an oxygen and glucose deprivation (OGD) model. Our data suggested that Myd88 was present in many retinal layers of sham-operated and ischemic mice, whereas Trif was mainly present in the ganglion cell layer (GCL). The level of Myd88 was increased in the retina after IR. We found that retinas of *Trif*KO mice had a significantly reduced neurotoxic pro-inflammatory response and significantly increased survival of the GCL neurons after IR. Although *Myd88*KO mice had relatively low levels of inflammation in ischemic retinas, their levels of IR-induced retinal damage were notably higher than those of *Trif*KO mice. We also found that Trif-deficient RGCs were more resistant to death induced by OGD than were RGCs isolated from *Myd88*KO mice. These data suggested that, as compared with the Myd88-dependent signaling cascade, Trif signaling contributes significantly to retinal damage after IR.

## Introduction

Retinal ischemia–reperfusion (IR) injury is a clinical condition that frequently causes blindness, owing to ineffective treatment (Osborne *et al*., 2004). Sterile inflammation, or an innate immune response in the absence of live pathogens, is an unavoidable consequence of retinal IR injury, which jeopardizes the viability of retinal ganglion cells (RGCs) at the site of injury (Dvoriantchikova *et al*., 2009, 2010a,b2010b, 2011; Chen & Nunez, 2010; Eltzschig & Eckle, 2011). Until recently, the molecular mechanisms that trigger an innate immune response in the retina after IR were poorly understood. However, we now know that endogenous ligands (damage-associated molecular patterns) liberated from necrotic cells act through pattern recognition receptors, initiating an innate immune response and thus affecting neuronal survival in ischemic tissue (Fujita *et al*., 2009; Dvoriantchikova *et al*., 2010a, 2011; Piccinini & Midwood, 2010). Toll-like receptors are a family of pattern recognition receptors that initiate an innate immune response in the presence of exogenous (pathogens) and endogenous (damage-associated molecular patterns) ligands (Janeway & Medzhitov, 2002; Piccinini & Midwood, 2010). Among toll-like receptors, Tlr4 is the most studied, and its critical role in IR-triggered damage and sterile inflammation in the retina has been acknowledged (Dvoriantchikova *et al*., 2010b; Ishizuka *et al*., 2013). The role of Tlr4-dependent signaling cascades in IR-induced retinal injury, however, has not been identified. Therefore, understanding the contribution of Tlr4-dependent signaling cascades in retinal IR injury will provide substantial insights and novel therapeutic strategies for this serious and difficult-to-treat condition.

Tlr4 signaling consists of two distinct signaling cascades, myeloid differentiation primary response 88(Myd88)-dependent and TIR-domain-containing adapter inducing interferon-β (Trif)-dependent, which facilitate the activation of nuclear factor-κB, leading to a pro-inflammatory response in the tissue (Janeway & Medzhitov, 2002; Takeda & Akira, 2005). In addition, the Trif-dependent signaling cascade mediates interferon-β production, which is associated with an anti-viral response (Janeway & Medzhitov, 2002; Takeda & Akira, 2005). It has also been shown that Tlr4 can directly trigger cell death in a Trif-dependent manner (He *et al*., 2011). Thus, Tlr4 can contribute to retinal damage after IR through induction of inflammatory responses and through the direct loss of cells. These effects in a cell may be dependent on whether Trif-dependent or Myd88-dependent signaling cascades are activated upon Tlr4 ligation. In this study, we evaluated the role of the Myd88-dependent and Trif-dependent signaling cascades in retinal damage triggered by IR, by using *Myd88* knockout (*Myd88*KO) and *Trif* knockout (*Trif*KO) mice. We found that mice lacking Trif had considerably less retinal damage following IR; therefore, RGC survival was increased and inflammation was decreased. However, although *Myd88*KO mice had low levels of inflammation in ischemic retinas, they had much higher damage after IR than *Trif*KO mice. We suggest that the Trif-dependent signaling cascade plays a deleterious role in retinal IR injury, whereas the role of the Myd88-dependent signaling cascade is not very noteworthy as compared with Trif-dependent signaling after IR.

## Materials and methods

### Animals

All experiments and postsurgical care were performed in compliance with the NIH Guide for the Care and Use of Laboratory Animals and with the Association for Research in Vision and Ophthalmology statement for the use of animals in ophthalmic and vision research, and according to the University of Miami Institutional Animal Care and Use Committee approved protocols. *Myd88*KO and *Trif*KO mice (stock numbers 009088 and 005037) and C57BL/6J (stock number 000664) mice, as the wild-type (WT) control, were obtained from the Jackson Laboratory (Bar Harbor, ME, USA). Mice were housed under standard conditions of temperature and humidity, with a 12-h light/dark cycle and free access to food and water. We used 1-month-old and 3-month-old male mice, or 3-day-old and 12-day-old pups. Mice were killed by CO_2_ inhalation under anesthesia.

### Transient retinal ischemia

Anesthesia was induced with isoflurane, and maintained for 45 min. Body temperature was held constant at 37 °C with a temperature-controlled heating pad. Retinal ischemia was induced for 45 min by introducing into the anterior chamber of the left eye a 33-gauge needle attached to a normal (0.9% NaCl) saline-filled reservoir, which was raised above the mouse to increase intraocular pressure (IOP) (increased to 120 mmHg). The contralateral eye was cannulated and maintained at normal IOP to serve as a normotensive control. Complete retinal ischemia, shown by whitening of the anterior segment of the eye and blanching of the retinal arteries, was verified by microscopic examination. After needle removal, erythromycin ophthalmic ointment (Fera Pharmaceuticals, Locust Valley, NY, USA) was applied to the conjunctival sac.

### Immunohistochemistry

For immunohistochemistry of the whole retina flatmounts, eyes were enucleated upon killing of the mouse, and immersion-fixed in 4% paraformaldehyde for 1 h; the retinas were then removed. The retinas were cryoprotected overnight in 30% sucrose, subjected to three freeze–thaw cycles, rinsed in 0.1 m Tris buffer, blocked for 1 h in buffer (5% donkey serum and 0.1% Triton X-100 in 0.1 m Tris buffer), and incubated overnight with fluorescein isothiocyanate-conjugated neuronal nuclei (NeuN) antibody (1 : 300; Chemicon, Billerica, MA, USA), Cy3-conjugated anti-glial fibrillary acidic protein (GFAP) antibody (1 : 150; Sigma-Aldrich, St Louis, MO, USA), and Cy5-conjugated anti-Cd11b antibody (1 : 150; Life Technologies, Grand Island, NY, USA). After being rinsed in 0.1 m Tris buffer, the retinas were flatmounted, coverslipped, and imaged with a Leica TSL AOBS SP5 confocal microscope (Leica Microsystems, Exton, PA, USA). To study Trif and Myd88 distribution in retinal layers, the fixed retinas were sectioned to a thickness of 100 μm with a vibratome (Leica Microsystems) and immunostained as described previously (Dvoriantchikova *et al*., 2009, 2012). Briefly, sections were permeabilized with 0.3% Triton X-100 in phosphate-buffered saline (PBS) for 1 h, rinsed three times in PBS, blocked in buffer (5% donkey serum, 2% bovine serum albumin and 0.15% Tween-20 in PBS) for 1 h, and incubated overnight with Trif-specific and Myd88-specific antibodies (both from GeneTex, Irvine, CA, USA), anti-Tubb3 antibody (β-tubulin III antibody, 1 : 500; Covance, Princeton, NJ, USA), and species-specific secondary fluorescent antibodies (Life Technologies). Control sections were incubated without primary antibodies. Imaging was performed with a Leica TSL AOBS SP5 confocal microscope (Leica Microsystems).

### Counting of ganglion cell layer (GCL) neurons

NeuN-positive neurons in the GCL were imaged by confocal microscopy in flatmounted retinas. Individual retinas were sampled randomly to collect a total of 20 images, from four retinal quadrants, with a ×20 objective lens. Five images were collected from each quadrant: one from the center, two from the middle, and two from the peripheral regions of the retina. NeuN-positive neurons were counted with imagej software. Cell loss in the ischemic retinas was calculated as a percentage of the mean cell density in normotensive fellow control eyes.

### Isolation of primary RGCs, astrocytes, and microglial cells

RGCs were isolated according to the two-step immunopanning protocol (Dvoriantchikova *et al*., 2011, 2012). Briefly, the whole retinas were incubated in papain solution (16.5 U/mL) for 30 min, and macrophages and endothelial cells were then removed from the cell suspension by panning with the anti-macrophage antiserum (Accurate Chemical, Westbury, NY, USA). In the next step, RGCs were bound to the panning plates containing anti-Thy1.2 antibody and released by trypsin incubation. Primary RGCs were cultured in Neurobasal/B27 medium (Life Technologies) 1 day before the experiment. Astrocytes and microglial cells were prepared from the brains of neonatal (postnatal day 3) mice as previously described (Dvoriantchikova *et al*., 2011; Barakat *et al*., 2012; Santos *et al*., 2014). These cells were cultured in Dulbecco's modified Eagle's medium (Life Technologies) containing 10% heat-inactivated fetal bovine serum (Life Technologies) and 1% antibiotic/antimycotic (Life Technologies).

### Oxygen and glucose deprivation (OGD) model

Primary RGCs isolated with a two-step immunopanning procedure were plated on poly(d-lysine)-treated and laminin-treated (both from Sigma-Aldrich) coverslips in 24-well plates, and cultured in medium (Neurobasal/B27; Life Technologies) 1 day before the experiment. Neurobasal/B27 medium was then replaced with ‘OGD medium’ containing 1.8 mm CaCl_2_, 0.814 mm MgCl_2_, 5.33 mm KCl, 26.19 mm NaHCO_3_, 68.97 mm NaCl, 0.906 mm NaH_2_PO_4_.H_2_O, and 10 mm sucrose (pH 7.4). The OGD medium was deoxygenated before the experiment by bubbling for at least 1 h with 95% N_2_/5% CO_2_. Primary RGC cultures were deprived of oxygen by use of an anaerobic chamber (5% CO_2_ and 95% N_2_) for 4 h at 37 °C. The OGD medium was then replaced with ‘sham medium’, which had the same composition, except that sucrose was replaced with 10 mm d-glucose, and cultures were returned to a normoxic environment. Parallel cultures were exposed to oxygenated sham medium in a normoxic incubator (37 °C; 5% CO_2_) to serve as controls.

### Cell death assay

After treatment, the apoptotic and necrotic neurons were labeled with the Vybrant Apoptosis Assay Kit (Life Technologies). Cells were imaged with a Leica TSL AOBS SP5 confocal microscope (Leica Microsystems). Individual glasses were sampled randomly to collect at least six images with a ×20 objective lens. The apoptotic and necrotic RGCs were counted semi-automatically with NIH imagej software. The percentages of live, apoptotic and necrotic RGCs relative to the total number of counted cells on the glass were determined. The experiment was repeated at least three times.

### Quantitative reverse transcription polymerase chain reaction (qRT-PCR) analysis

Quantitative RT-PCR analysis was performed with gene-specific primers (Table1). Specifically, total RNA was extracted from retinas or primary cells (RGCs, astrocytes, and microglia) with Absolutely RNA Nanoprep or Microprep kits (Agilent Technologies, Santa Clara, CA, USA), and reverse transcribed with the Reverse Transcription System (Promega, Madison, WI, USA) to synthesize cDNA. To quantify the absolute number of transcripts of a gene of interest in cDNA samples, we used a standard curve. We prepared six standards, in which a gene of interest was present at 2 × 10^5 ^copies, 2 × 10^4 ^copies, 2 × 10^3 ^copies, 2 × 10^2 ^copies, 20 copies, and two copies. We used a combination of 5 μL of tested cDNA samples or standards with the SYBR GREEN PCR MasterMix (Qiagen, Valencia, CA, USA) and gene-specific primers to perform qRT-PCR in the Rotor-Gene Q Cycler (Qiagen). Quantitative RT-PCR growth curves for standards and tested cDNA samples for the same gene were used to prepare a standard curve and calculate the absolute number of gene transcripts with the Rotor-Gene Q Cycler software. The absolute number of the studied gene transcripts obtained was divided by the absolute number of housekeeping gene [β-actin (*Actb*) or succinate dehydrogenase subunit A (*Sdha*)] transcripts in the same cDNA sample. These normalized data were used for further analysis.

**Table 1:** List of PCR primers

Gene	Oligonucleotides, 5′–3′	
*Tlr1*	Forward	ATGCACAGCTCCTTGGTTTT
	Reverse	GTCCTTGGGCACTCTGGTAA
*Tlr2*	Forward	CGGACTGTTTCCTTCTGACC
	Reverse	GAGTCCGGAGGGAATAGAGG
*Tlr3*	Forward	GAGCCAGAACTGTGCCAAAT
	Reverse	CGCAACGCAAGGATTTTATT
*Tlr4*	Forward	CAGCAAAGTCCCTGATGACA
	Reverse	GTCTCCACAGCCACCAGATT
*Tlr6*	Forward	CCGTCAGTGCTGGAAATAGAG
	Reverse	ATATCAGGCATCCGAAGCTC
*Tlr7*	Forward	GGCTCCCTTCTCAGGATGAT
	Reverse	CCCTCAGGGATTTCTGTCAA
*Tlr8*	Forward	AAACAAACGTTTTACCTTCCTTTG
	Reverse	TCGTTATATGTGTAATGGCATTGTC
*Tlr9*	Forward	CCATCTCCCAACATGGTTCT
	Reverse	GTTGGACAGGTGGACGAAGT
*Myd88*	Forward	CTTGATGACCCCCTAGGACA
	Reverse	AGGCTGAGTGCAAACTTGGT
*Trif*	Forward	AGACCCCTACAGCCAGGTCT
	Reverse	GGCATGGAGAAGCTTTGACT
*Il1b*	Forward	GACCTTCCAGGATGAGGACA
	Reverse	AGGCCACAGGTATTTTGTCG
*Tnf*	Forward	CAAAATTCGAGTGACAAGCCTG
	Reverse	GAGATCCATGCCGTTGGC
*Ccl2*	Forward	AGGTCCCTGTCATGCTTCTG
	Reverse	ATTTGGTTCCGATCCAGGTT
*Ccl5*	Forward	AGCAGCAAGTGCTCCAATCT
	Reverse	ATTTCTTGGGTTTGCTGTGC
*Cxcl10*	Forward	GCTGCAACTGCATCCATATC
	Reverse	CACTGGGTAAAGGGGAGTGA
*Icam1*	Forward	TGGTGATGCTCAGGTATCCA
	Reverse	CACACTCTCCGGAAACGAAT
*Nox2*	Forward	GACTGCGGAGAGTTTGGAAG
	Reverse	ACTGTCCCACCTCCATCTTG
*Nos2*	Forward	CAGAGGACCCAGAGACAAGC
	Reverse	TGCTGAAACATTTCCTGTGC
*Thy1*	Forward	CGCTCTCCTGCTCTCAGTCT
	Reverse	ATCCTTGGTGGTGAAGTTGG
*Gfap*	Forward	AGAAAGGTTGAATCGCTGGA
	Reverse	CGGCGATAGTCGTTAGCTTC
*Cd11b*	Forward	CCATCCCATCTTTCCTGCTA
	Reverse	GGATGATCCCATACGGTCAC
*Sdha*	Forward	ACACAGACCTGGTGGAGACC
	Reverse	GCACAGTCAGCCTCATTCAA
*Actb*	Forward	CACCCTGTGCTGCTCACC
	Reverse	GCACGATTTCCCTCTCAG

### Statistical analysis

Statistical analysis was performed with a one-way anova or Student's *t*-test. *P*-values of ≤ 0.05 were considered to be statistically significant.

## Results

### Trif and Myd88 show opposite patterns of expression in the retina and RGCs after IR

**Figure 1:**
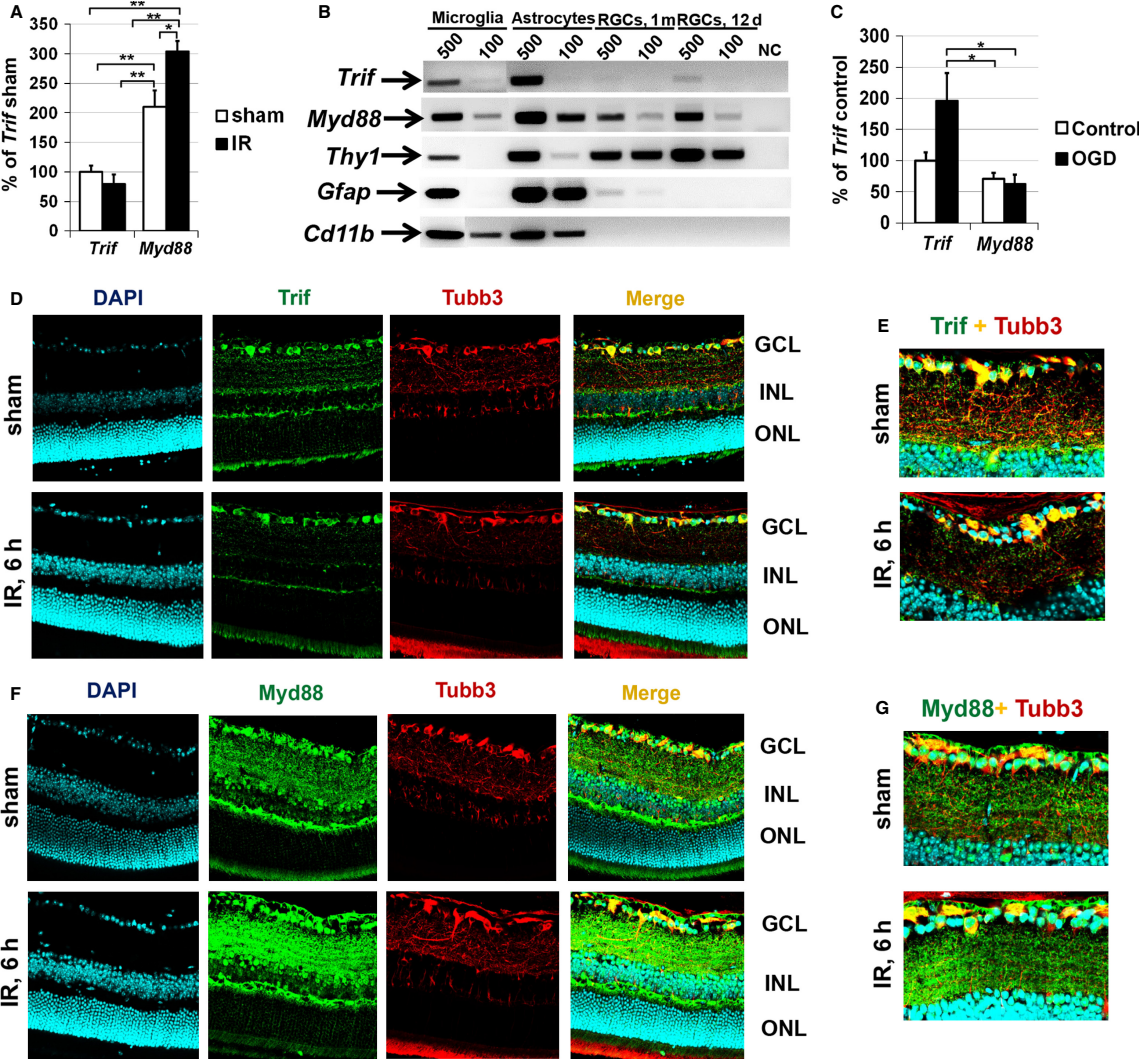
Myd88 is mainly present in retinas of sham-operated and ischemic mice, whereas Trif is mainly present in the GCL and RGCs. (A) Expression of *Trif* and *Myd88* in sham-operated and ischemic retinas 6 h after reperfusion was evaluated by qRT-PCR. For each gene, results are expressed as a percentage of the corresponding value for *Trif* expression in the sham-operated retina ± SE of the mean (**P < 0.01, *P < 0.05, *n* = 6). (B) Micrograph of the gel-separated qRT-PCR products for *Trif* and *Myd88* obtained by the use of total RNA isolated from 500 and 100 microglial cells, astrocytes, and RGCs. RGCs were purified with a two-step immunopanning protocol from 12-day-old and 1-month-old mice. The purity of isolated RGCs was verified by testing the presence of the RGC marker (*Thy1*), as well as marker genes for astrocytes (*Gfap*) and microglial cells (*Cd11b*). (C) Differential expression of *Trif* and *Myd88* in control RGCs and RGCs subjected to OGD followed by 24 h of re-oxygenation. (D–G) Immunohistochemistry showed accumulation of Myd88 in many retinal layers of sham-operated and ischemic mice 6 h after reperfusion. Trif is mainly present in the GCL. Antibodies against tubulin β-III (Tubb3) were used to identify RGCs. 4′,6-Diamidino-2-phenylindole was used to label DNA, and thus allowed visualization of the nucleus of the cell. INL, inner nuclear layer; ONL, outer nuclear layer.

As stimulation of Tlr4 facilitates the activation of two distinct signaling cascades, Trif-dependent and Myd88-dependent, Tlr4-mediated effects in the cell depend on the presence and proportion of Trif and Myd88. To evaluate the presence of Trif and Myd88 in the retina in normal and pathological conditions, we first tested *Trif* and *Myd88* expression in sham-operated and ischemic retinas. Retinal ischemia was induced by unilateral elevation of IOP for 45 min, and retinas were collected 6 h post-reperfusion. Total RNA was isolated from sham-operated and ischemic retinas, and subjected to qRT-PCR analysis with *Trif*-specific and *Myd88*-specific oligonucleotides. We found that expression of *Myd88* was upregulated in ischemic retinas, whereas the expression of *Trif* was not altered (Fig.1A). As the Trif-dependent and Myd88-dependent signaling cascades can affect cell death and survival, we also evaluated the expression of *Trif* and *Myd88* in RGCs. RGCs were isolated from retinas of 12-day-old pups and 1-month-old mice by a two-step immunopanning procedure, immediately lysed after purification, and tested by qRT-PCR for the presence of *Trif* and *Myd88* transcripts. We found expression of *Myd88* and *Trif* in RGCs (Fig.1B). The purity of isolated RGCs was verified by testing the presence of the marker genes for RGCs (*Thy1*), astrocytes (*Gfap*), and microglial (*Cd11b*) cells (Fig.1B). Primary astrocytes and microglial cells were used as positive controls (Fig.1B). The level of contamination was insufficient to affect our conclusions regarding the presence of Trif and Myd88 in isolated RGCs. Next, we evaluated *Trif* and *Myd88* expression in primary RGC cultures subjected to OGD. The same quantity of RGCs isolated by immunopanning from retinas of 12-day-old pups was plated on 24-well plates and, 24 h later, RGC cultures were deprived of oxygen and glucose for 4 h in an anaerobic chamber. After OGD, the OGD medium was replaced with sham medium (see Materials and methods), and cultures were returned to a normoxic environment. Parallel cultures were exposed to oxygenated sham medium in a normoxic incubator (37 °C; 5% CO_2_) to serve as controls. RGCs were lysed 24 h after re-oxygenation, and used in qRT-PCR analysis. We found that the expression of *Trif* in control and OGD-subjected RGCs was significantly higher than the expression of *Myd88* (Fig.1C). Our data also indicated increased expression of *Trif* after OGD.

Next, the spatial distribution of Trif and Myd88 was evaluated by immunohistochemistry in fixed retinas of sham-operated and ischemic mice 6 h post-reperfusion. Trif-specific immunostaining was evident predominantly in the GCLs of sham-operated and ischemic retinas (Fig.1D), which contain RGCs and displaced amacrine cells. As shown in Fig.1D and E, substantial Trif-specific immunostaining was localized to the somata of GCL neurons in the sham-operated and ischemic retinas. Importantly, double staining with an RGC marker (Tubb3) suggested that Trif-positive GCL neurons were RGCs (Fig.1D and E). At the same time, our data indicated the presence of Myd88 throughout the nerve fiber layer (contains astrocytes and microglial cells), the GCL, the inner plexiform layer, and the inner nuclear layer (Fig.1F). Myd88 was present in the body and processes of the GCL neurons (Fig.1F and G). Our data also suggested that the level of Myd88 was higher in ischemic retinas than in sham-operated controls (Fig.1F and G). Control immunohistochemical data, obtained with retinal sections from *Trif*KO and *Myd88*KO mice, demonstrated the specificity of anti-Trif and anti-Myd88 antibodies (Fig. S1). Negative controls incubated with secondary antibody only did not show any specific immunostaining (data not shown). These findings indicate that the immunohistochemical results for Trif and Myd88 accumulation and levels in the sham-operated and ischemic retinas were consistent with our qRT-PCR data above. Thus, the Myd88-dependent signaling cascade can be activated in many retinal cell types (neurons and glial cells), whereas the level of Trif was significantly higher in RGCs.

### TrifKO mice showed significant neuroprotection as compared with Myd88KO mice after IR

**Figure 2:**
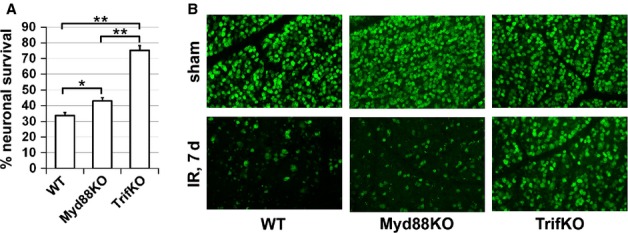
Suppression of Trif-dependent and Myd88-dependent signaling activity promotes neuroprotection in the GCL after IR. (A) The percentage of GCL neuron survival in retinas of WT,*Trif*KO and *Myd88*KO mice 7 days after IR, determined by bilateral comparison for individual mice (**P < 0.01, *P < 0.05, *n* = 5 mice per group). (B) Representative confocal images of NeuN-labeled GCLs (green) in flatmounted retinas in sham-operated controls and ischemic retinas 7 days after reperfusion.

To evaluate the effects of the Myd88-dependent and Trif-dependent signaling cascades on the severity of retinal ischemia, we used *Myd88*KO and *Trif*KO mice. *Myd88*KO and *Trif*KO mice were indistinguishable from WT mice in every respect (retinal morphology, total number of GCL neurons, etc.), showing no phenotypic abnormalities. Retinal ischemia was induced for 45 min in WT, *Myd88*KO and *Trif*KO mice. The contralateral eyes were used as sham-operated normotensive controls. The experimental and sham-operated retinas were collected 7 days after reperfusion. Whole retina flatmounts were stained for the neuronal marker NeuN to quantify the number of surviving neurons in the GCL of ischemic and control retinas. Cell loss in the ischemic retinas was calculated as a percentage of the mean cell density in the normotensive fellow control eye. We found significant IR-induced loss of retinal neurons in WT retinas (33 ± 2%), whereas *Trif*KO ischemic retinas showed significant neuronal survival (75 ± 3%) (Fig.2). We also found a statistically significant difference in GCL neuronal survival between WT and *Myd88*KO ischemic mice (Fig.2). However, this difference was not large (*Myd88*KO mice, 43 ± 2%; WT mice, 33 ± 2%; *P* < 0.05) in comparison with the GCL neuronal survival difference found between *Trif*KO and WT mice (75 ± 3% vs. 33 ± 2%, *P* < 0.01) (Fig.2). Thus, Trif deficiency resulted in neuroprotective effects in the GCL of retinas after IR, whereas the Myd88 effect was not found to be very significant.

### Inactivation of the Trif-dependent and Myd88-dependent signaling cascades resulted in reduced inflammation following retinal ischemia

**Figure 3:**
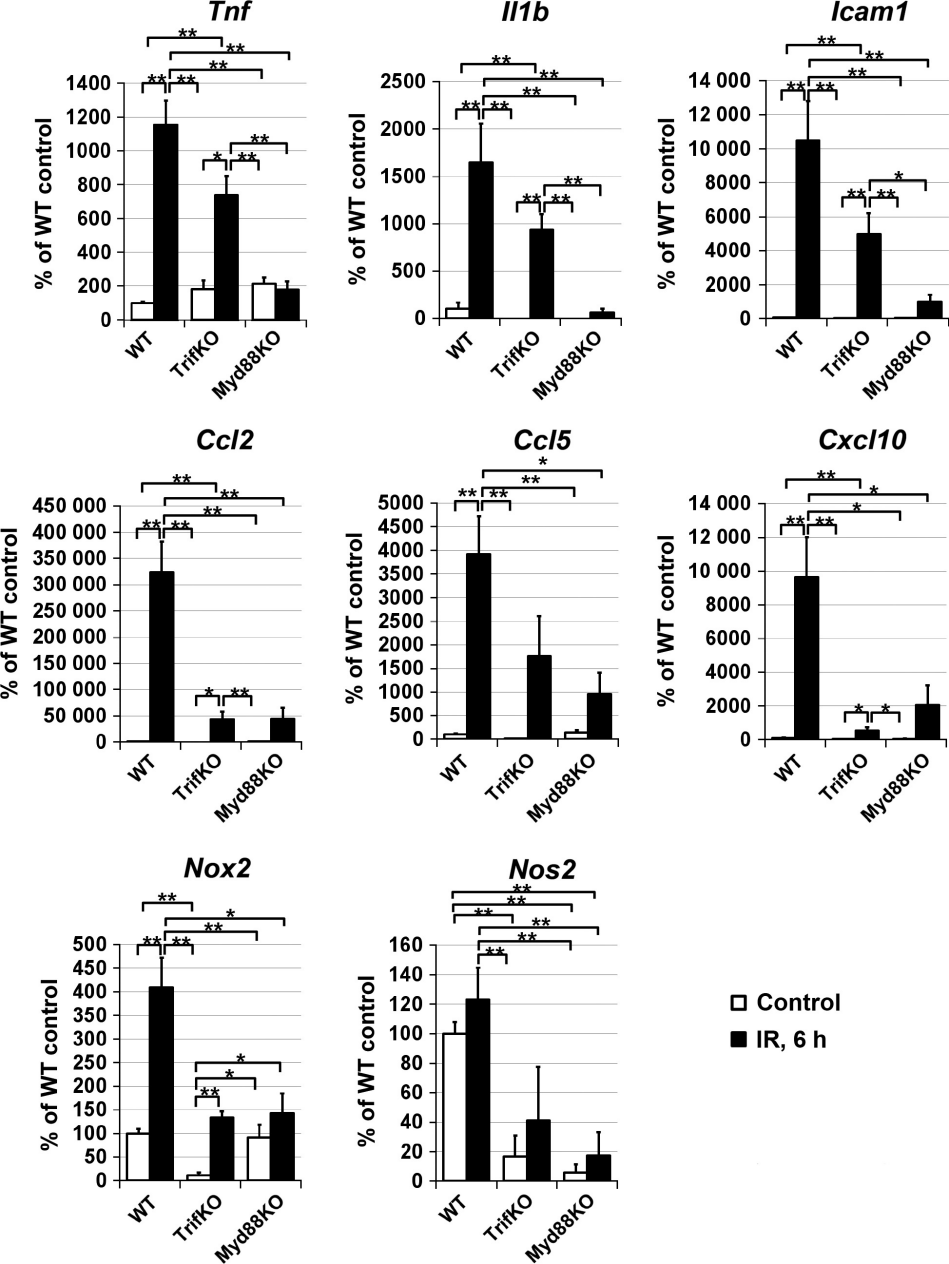
Trif and Myd88 deficiency suppresses the induction of pro-inflammatory markers after IR. Gene expression was assessed by qRT-PCR in sham-operated controls and experimental retinas of WT,*Trif*KO and*Myd88*KO mice 6 h after IR. For each gene, results are expressed as a percentage of the corresponding value in the sham-operated retina ± SE of the mean (**P < 0.01, *P < 0.05, *n* = 6 mice per group).

The Trif-dependent and Myd88-dependent signaling cascades are key players in Tlr-mediated inflammation in tissue (Janeway & Medzhitov, 2002; Takeda & Akira, 2005). Thus, to study the molecular changes associated with the elevated resistance to retinal IR injury in *Trif*KO and *Myd88*KO mice, we compared the expression of several genes known to be involved in the IR-induced pro-inflammatory response in ischemic tissue. We measured gene expression in total RNA extracted from the whole retina 6 h post-reperfusion, because most changes in gene expression for pro-inflammatory factors typically occur shortly after IR injury. Transcriptional upregulation of cytokines (*Thf* and *Il1b*), chemokines (*Ccl2*,*Ccl5*, and *Cxcl10*), a cell adhesion molecule (*Icam1*), inducible nitric oxide synthase (*Nos2*), and the Nox2 gene(*Cybb*), encoding the catalytic subunit of the NADPH oxidase protein complex, was evident in all ischemic retinas of WT and knockout mice 6 h after reperfusion (Fig.3). In *Myd88*KO and*Trif*KO mice, however, the expression of *Tnf*,*Il1b*,*Ccl2*,*Ccl5*,*Cxcl10*, *Icam1*, *Nos2* and *Nox2* was significantly reduced relative to WT mice. We also noted that retinas from experimental eyes of *Myd88*KO mice had significantly lower levels of cytokine (*Thf* and *Il1b*) and cell adhesion molecule (*Icam1*) expression than *Trif*KO mice (Fig.3).

**Figure 4:**
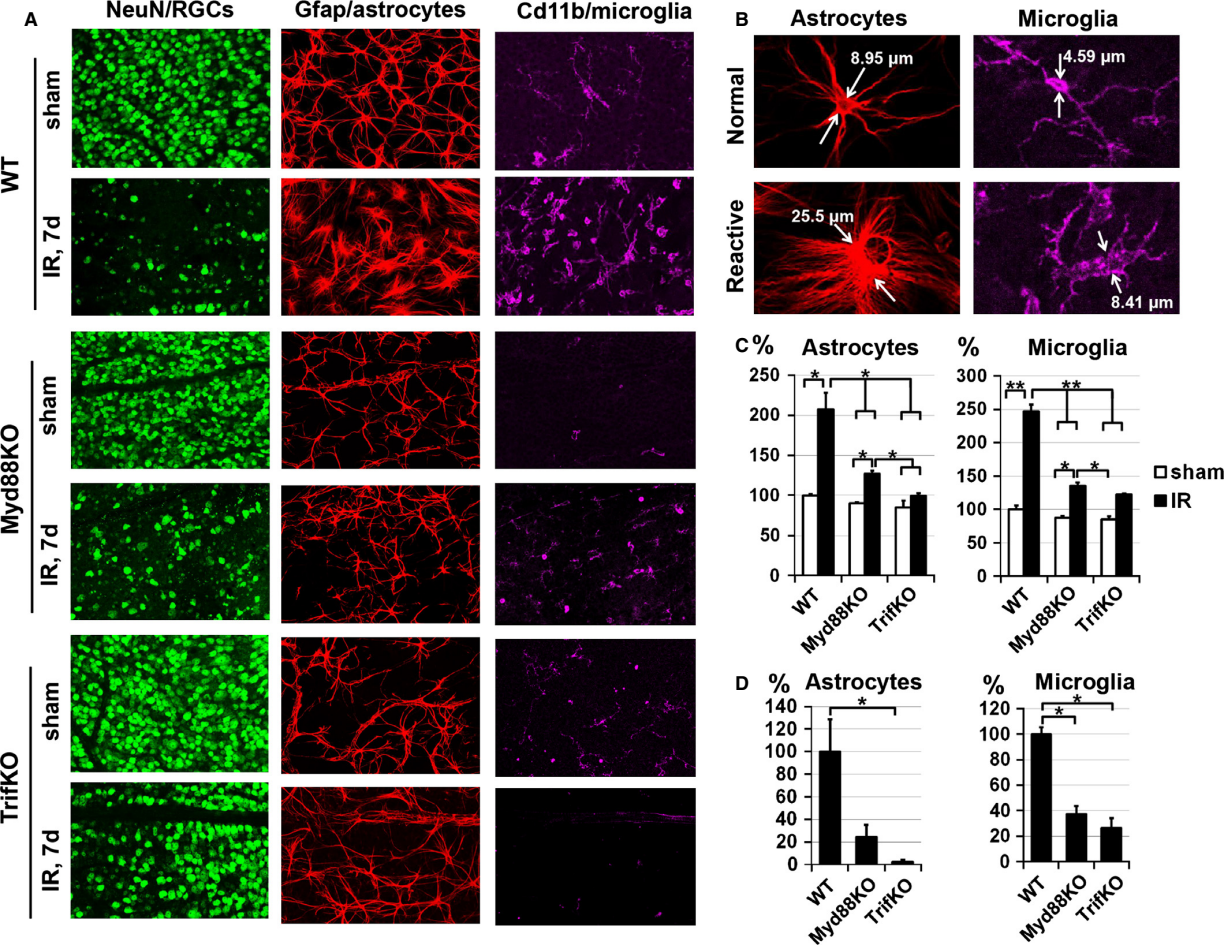
Trif and Myd88 deficiency suppresses reactive gliosis in retinas after IR. (A) Astrocyte (GFAP-labeled, red) and microglial (Cd11b-labeled, purple) cell morphology assessed in flatmounted retinas was similar in sham-operated WT,*Trif*KO and *Myd88*KO retinas, but changed dramatically in post-ischemic WT retinas 7 days after reperfusion, showing significant hypertrophy. Significantly altered cells were located in the areas of greatest damage [reduced number of NeuN-labeled (green) neurons]. In contrast, the morphology of *Trif*KO and *Myd88*KO glial cells (astrocytes and microglia) changed insignificantly following IR. (B) These confocal images represent typical glial cells, which we recognized as normal and reactive astrocytes and microglial cells, on the basis of size and morphology. (C) Individual retinas from sham-operated and ischemic eyes of WT,*Trif*KO and *Myd88*KO mice were sampled randomly to collect a total of 20 images, from four retinal quadrants. Sizes of individual astrocytes and microglial cells in these images were measured and averaged. The final results are expressed as a percentage of the average size of glial cells in the retinas of the sham-operated eye ± SE of the mean of WT mice (**P < 0.01, *P < 0.05). (D) The quantity of glial cells with the characteristics of reactive glial cell morphology was determined in sham-operated and IR retinas of WT,*Trif*KO and *Myd88*KO mice. The results are expressed as a percentage of the average quantity of reactive glial cells in the retinas of WT mice (*P < 0.05).

As strong reactive gliosis is a hallmark of an increased innate immune response in ischemic tissue in the central nervous system (Clemens *et al*., 1996; Dvoriantchikova *et al*., 2009; Lu *et al*., 2011; Barakat *et al*., 2012), we tested the reactive state of glial cells in ischemic retinas of WT, *Myd88*KO and *Trif*KO mice. Retinal IR was induced in WT, *Myd88*KO and *Trif*KO mice, and the types of glial cell were investigated in flatmounted retina 7 days after reperfusion by immunohistochemistry with cell type-specific markers (GFAP as a marker of astrocytes, Cd11b as a marker of microglial cells, and NeuN as a marker of retinal neurons). Glial (astrocytes and microglia) hypertrophy is a hallmark of reactive gliosis in tissue (Clemens *et al*., 1996; Dvoriantchikova *et al*., 2009; Lu *et al*., 2011; Barakat *et al*., 2012). We observed a higher number of hypertrophic GFAP-positive and Cd11b-positive cells with less ramified processes in WT than in *Myd88*KO and *Trif*KO ischemic retinas (Fig.4A). To quantitatively evaluate glial reactivity, we measured average glial cell size (Fig.4B) and the average quantity of glial cells with the characteristics of reactive glial cell morphology (Fig.4B) in sham-operated and IR retinas of WT, *Trif*KO and *Myd88*KO mice. We found that average glial cell size and the quantity of reactive glial cells were higher in IR retinas of WT mice than in retinas of *Trif*KO and *Myd88*KO mice (Fig.4C and D). Collectively, these data suggest that the IR-induced pro-inflammatory response in *Myd88*KO and *Trif*KO retinas was significantly reduced relative to that in WT retinas.

### RGCs from TrifKO mice are more resistant to death induced by OGD than RGCs from Myd88KO mice

**Figure 5:**
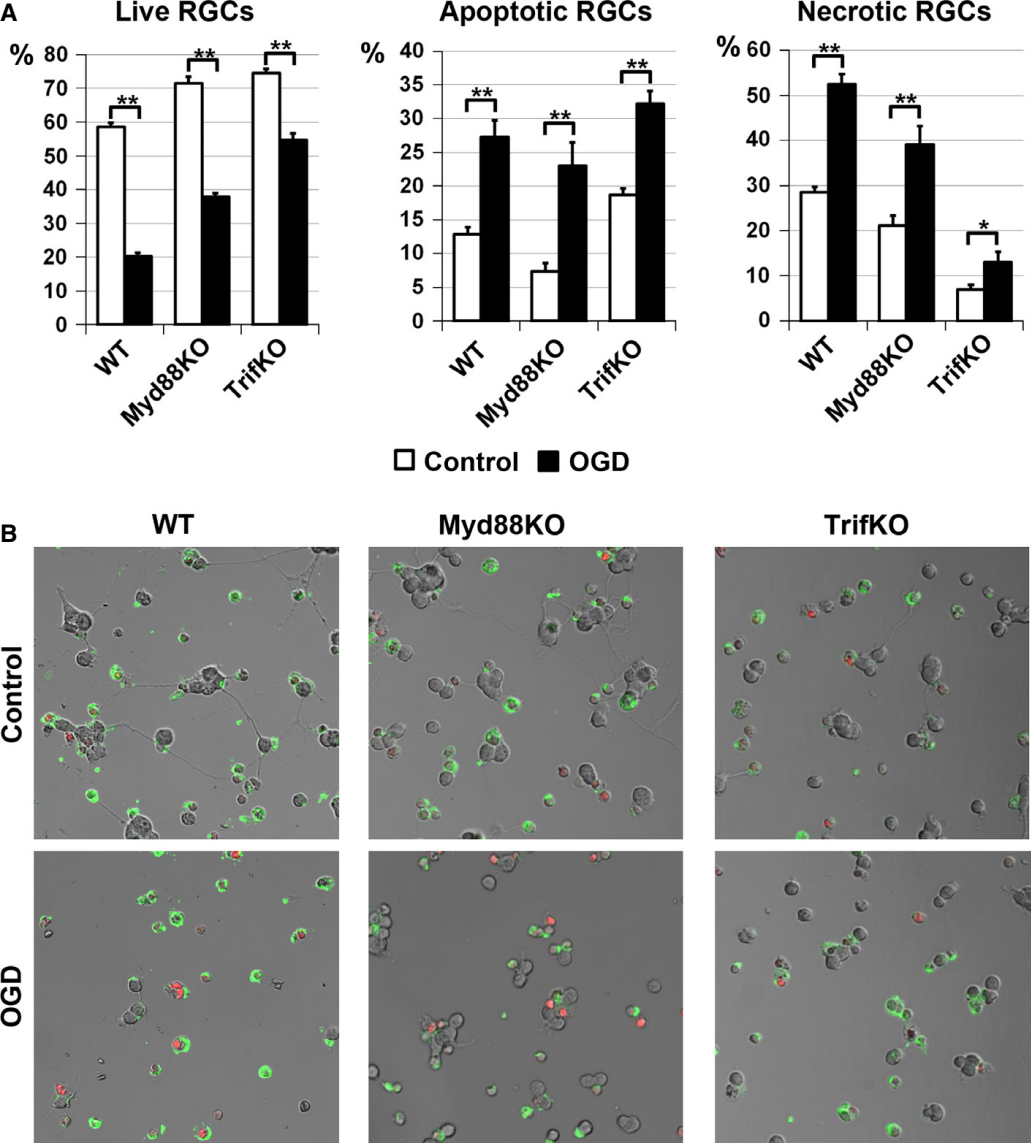
Suppression of Trif-dependent and Myd88-dependent signaling activity in RGCs promotes neuronal survival after OGD. RGCs isolated from WT,*Trif*KO and *Myd88*KO mice were deprived of oxygen and glucose for 4 h, and this was followed by 24 h of re-oxygenation (control, **P < 0.01, *P < 0.05). (A) The percentages of living apoptotic and necrotic RGCs relative to the total number of counted cells on the same coverslip were calculated. (B) Representative confocal images. Apoptotic RGCs were identified with annexin V (green) as a marker of apoptotic cells. To recognize necrotic RGCs, we used propidium iodide (red) as a marker for cells that have lost membrane integrity (necrosis).

As the neurotoxic pro-inflammatory response was reduced in retinas of both *Myd88*KO and *Trif*KO mice after IR, but neuroprotection was higher in *Trif*KO retinas than in *Myd88*KO retinas, we thought that the neuronal Trif-dependent signaling cascade might play an important role in mediating IR-induced RGC death. To test this hypothesis, we used primary RGC cultures. The RGCs isolated from WT, *Trif*KO and *Myd88*KO mice were plated on coverslips and, 24 h later, these RGC cultures were subjected to OGD for 4 h in an anaerobic chamber. Parallel cultures exposed to oxygenated sham medium were used as controls. The quantities of both live RGCs and RGCs undergoing apoptosis and necrosis were determined 24 h after re-oxygenation, by use of the Vybrant Apoptosis Assay Kit. We determined the percentages of living necrotic and apoptotic RGCs relative to the total number of counted cells on the same coverslip. We found that, whereas RGCs isolated from WT mice and subjected to OGD showed high levels of cell death, predominantly by necrosis (live RGCs, 20 ± 1%; apoptotic RGCs, 28 ± 3%; necrotic RGCs, 52 ± 2%; Fig.5), Trif and Myd88 deficiency significantly increased RGC survival and decreased necrotic cell death following OGD (*Trif*KO, live RGCs, 55 ± 2%; *Trif*KO, apoptotic RGCs, 32 ± 2%; *Trif*KO, necrotic RGCs, 13 ± 2%; *Myd88*KO, live RGCs, 38 ± 1%; *Myd88*KO, apoptotic RGCs, 23 ± 4%; *Myd88*KO, necrotic RGCs, 39 ± 4%). At the same time, RGCs isolated from *Trif*KO mice showed maximal survival and minimal necrotic cell death as compared with RGCs isolated from *Myd88*KO mice. Interestingly, OGD-treated RGCs isolated from *Trif*KO mice predominantly underwent apoptosis, as opposed to RGCs isolated from WT and *Myd88*KO mice (Fig.5). Thus, the Trif-dependent signaling cascade can directly contribute to retinal damage after IR through loss of RGCs, primarily by necrosis.

### The retina and RGCs express toll-like receptors under normal and ischemic conditions

**Figure 6:**
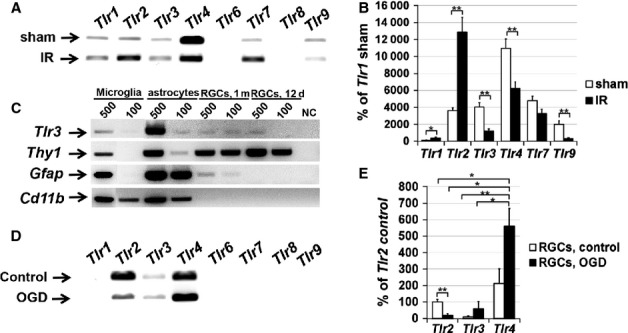
Toll-like receptors have broad expression in the retina. (A) Micrograph of the gel-separated PCR products from the RT-PCR test showing specific bands corresponding to mRNA for *Tlr1*,*Tlr2*,*Tlr3*,*Tlr4*,*Tlr7* and *Tlr9* detected in the retinas of sham-operated (sham) mice and in the ischemic retinas. (B) Differential expression of TLRs in sham-operated and ischemic retinas 6 h after reperfusion was tested by quantitative RT-PCR. For each gene, results are expressed as a percentage of the corresponding value for *Tlr1* expression in the sham-operated retina ± SE of the mean (**P < 0.01, *P < 0.05, *n* = 6). (C) Micrograph of the gel-separated PCR products from the qRT-PCR test showing specific bands corresponding to mRNA for *Tlr3*, *Thy1* (RGC marker), *Gfap* (astroglial marker) and *Cd11b* (microglial marker) detected in the total RNA samples isolated from 500 and 100 microglial cells, astrocytes, and RGCs. RGCs were purified with a two-step immunopanning protocol from 12-day-old and 1-month-old mice. (D) Micrograph of the gel-separated qRT-PCR products for the members of the toll-like receptor family obtained by using total RNA isolated from control and OGD-treated primary RGC cultures. (E) Relative enrichment of *Tlr2*, *Tlr3* and *Tlr4* transcripts detected by qRT-PCR in control and OGD-treated RGCs (**P < 0.01, *P < 0.05, *n* = 6).

Tlr4 is not the only member of the toll-like receptor family that can activate the Trif-dependent and Myd88-dependent signaling cascades. Whereas the Myd88-dependent signaling cascade is activated by all toll-like receptors (except for Tlr3), the Trif-dependent signaling cascade is only activated after ligation of Tlr3 and Tlr4 (Janeway & Medzhitov, 2002; Takeda & Akira, 2005). Thus, the observed Trif-dependent and Myd88-dependent effects in the retina after IR can be mediated after ligation of some other toll-like receptors. To establish which toll-like receptors may be involved, we decided to test the expression of various members of the toll-like receptor family in the retina and RGCs after IR. Retinal ischemia was induced, and the changes in the expression of toll-like receptors (*Tlr1–Tlr9*) were assessed 6 h post-reperfusion by qRT-PCR. We found that sham-operated and ischemic retinas expressed *Tlr1*, *Tlr2*, *Tlr3*, *Tlr4*, *Tlr7*, and *Tlr9* (Fig.6A and B). *Tlr6* and *Tlr8* transcripts were not detected in ischemic and sham-operated retinas. Whereas the expression levels of *Tlr1* and *Tlr2* were increased in ischemic retinas, the expression levels of *Tlr3*, *Tlr4* and *Tlr9* were reduced 6 h after reperfusion (Fig.6B). We found no significant differences in the level of *Tlr7* expression between ischemic and control retinas (Fig.5B). We also tested the expression of toll-like receptors in RGCs that were isolated from WT mice and immediately lysed after purification. These cells were tested by qRT-PCR for the presence of toll-like receptor gene transcripts. We found only expression of *Tlr3* in RGCs (Fig.6C). Next, we evaluated the expression of toll-like receptors in primary RGC cultures subjected to OGD. RGCs were lysed 24 h after re-oxygenation and used for qRT-PCR analysis. We found that, after culture of RGCs, in addition to *Tlr3* the RGCs started to also express *Tlr2* and *Tlr4* (Fig.6D). Measurement of the absolute expression of *Tlr2*,*Tlr3* and *Tlr4* in control and OGD-treated RGCs by qRT-PCR revealed that the *Tlr4* expression level was significantly higher than the *Tlr2* and *Tlr3* expression levels (Fig.6E). These data suggest that RGCs can initiate *Tlr2*,*Tlr3* and *Tlr4* expression under conditions of stress, such as IR. Finally, our findings indicate that many toll-like receptors are present in normal and ischemic retinas, and can therefore activate the Trif-dependent and Myd88-dependent signaling cascades.

## Discussion

Retinal IR injury is a common cause of blindness and visual impairment, seriously affecting the lives of people around the globe (Osborne *et al*., 2004). Thus, understanding the mechanisms of IR injury will provide substantial insights and novel therapeutic potential to regulate disease pathogenesis in the retina. Previously, we demonstrated the deleterious role of the Tlr4-dependent signaling cascade in retinal damage and inflammation triggered by IR (Dvoriantchikova *et al*., 2010b). However, the contribution of Tlr4-dependent signaling cascades, Trif and Myd88, in IR-induced retinal inflammation and damage remains unknown. In this study, we investigated the role of the Trif-dependent and Myd88-dependent signaling cascades in retinal IR injury. We found that the Trif-dependent signaling cascade promotes tissue damage after IR. At the same time, the level of retinal damage in *Myd88*KO mice after IR was lower than in WT mice, but significantly higher than in *Trif*KO mice. As both the Trif-dependent and Myd88-dependent signaling cascades can mediate a cytotoxic pro-inflammatory response in tissues, we thought that the observed difference in the level of retinal damage might be determined by the difference in the intensity of the pro-inflammatory activity in the retina after IR. Surprisingly, whereas the IR-induced pro-inflammatory response in *Myd88*KO and *Trif*KO mice was considerably lower than in WT mice, *Myd88*KO and *Trif*KO mice showed no difference in the levels of IR-induced inflammation. Concurrently, we found that the Trif-dependent signaling cascade plays a significant role in IR-induced retinal neuronal (RGC) death, unlike the Myd88-dependent signaling cascade. These data suggest that, unlike the Myd88-dependent signaling cascade, the Trif-dependent signaling cascade contributes directly to retinal damage after IR through loss of RGCs, and indirectly through induction of a neurotoxic pro-inflammatory response.

A large body of evidence suggests that Tlr4 plays a central role in IR injuries of many tissues (Wu *et al*., 2007; Pulskens *et al*., 2008; Arumugam *et al*., 2009; Dvoriantchikova *et al*., 2010b; Fang & Hu, 2011; Li *et al*., 2011; Altemeier *et al*., 2013). However, whereas the role of pathogen-induced Tlr4-dependent signaling (Trif and Myd88) is well understood (Janeway & Medzhitov, 2002; Takeda & Akira, 2005), there are many contradictory reports regarding the role of the Trif-dependent and Myd88-dependent signaling cascades in IR-induced inflammation and damage. Although there are reports that Myd88 and Trif deficiencies do not protect against IR injury (Pulskens *et al*., 2008; Hua *et al*., 2009), many other reports indicate a pivotal role of either the Myd88-dependent or the Trif-dependent signaling cascade, or both cascades, in inflammation and damage in tissue affected by IR (Wu *et al*., 2007; Victoni *et al*., 2010; Fang & Hu, 2011; Li *et al*., 2011; Altemeier *et al*., 2013; Stridh *et al*., 2013; Chen *et al*., 2014). Our data indicate that the Trif-dependent signaling cascade plays a critical role in IR-induced retinal inflammation and damage, whereas the Myd88-dependent signaling cascade does not. These data are in agreement with the results obtained by Lin *et al*., (2012) who demonstrated that Trif deficiency increases RGC survival after optic nerve crush (Lin *et al*., 2012). However, whereas the data of Lin *et al*. (2012) indicate the critical role of microglial Trif signaling, our results indicate that activity of the Trif-dependent signaling cascade in glial cells (astrocytes and microglia) and RGCs mediates retinal damage after IR. We also found that both *Trif*KO and *Myd88*KO mice showed significantly reduced pro-inflammatory responses in the retina after IR. Our data revealed that deficiency in the Myd88-dependent and Trif-dependent signaling cascades significantly reduced the levels of cell adhesion molecules and chemokines. We also detected decreased levels of catalytic subunits of enzymes producing reactive oxygen and nitrogen species. Levels of cytokines were also significantly lower in *Myd88*KO and *Trif*KO mice than in WT mice. Collectively, all of these factors can mediate neuronal toxicity, which has been observed previously (Yoneda *et al*., 2001; Berger *et al*., 2008; Li *et al*., 2011; Barakat *et al*., 2012; Altemeier *et al*., 2013). As we did not detect substantial differences in the levels of retinal inflammation between *Trif*KO and *Myd88*KO mice, we determined that a reduced pro-inflammatory response was not the only factor responsible for neuroprotection in the *Trif*KO retina after IR. It has previously been shown that Tlr4 (as well as Tlr3), owing to Trif-dependent signaling, can directly mediate necrotic cell death (He *et al*., 2011). As our data and the results of Ishizuka *et al*. (2013) indicate the presence of Tlr3 and Tlr4 in ischemic RGCs, we investigated whether Trif signaling facilitates retinal neuronal (RGC) death after IR. We found that the Trif-dependent signaling cascade can directly contribute to retinal damage after IR through the direct loss of RGCs, chiefly by necrosis. Interestingly, we also found that RGCs isolated from *Myd88*KO mice were resistant to death induced by OGD. This effect, however, was less significant than the effects on RGCs isolated from *Trif*KO mice. Collectively, these data can explain the meaningful neuroprotection in the retinas of *Trif*KO mice after IR.

In conclusion, our study has revealed, for the first time, that the Trif-dependent signaling cascade plays a deleterious role in retinal IR injury, whereas the role of the Myd88-dependent signaling cascade in retinal IR injury is not nearly as significant. Our data, as well as other published data, indicate that it is impossible to extrapolate the roles of the Trif-dependent and Myd88-dependent signaling cascades in IR-induced damage from one tissue to another. The available evidence appears to indicate that the effects of IR-induced, Trif signaling-mediated and Myd88 signaling-mediated damage are tissue-specific, and should be carefully investigated. Our study allowed us to establish the roles of the Trif-dependent and Myd88-dependent signaling cascades in retinal IR injury. Thus, as treatment for retinal IR injury is limited, in part because of a lack of understanding of the molecular events leading to neuronal damage after IR, a greater understanding of the roles of the Trif-dependent and Myd88-dependent signaling cascades in retinal IR injury may aid in the development of specific therapies to treat this condition.

## Abbreviations

GCL: ganglion cell layer
GFAP: glial fibrillary acidic protein
IOP: intraocular pressure
IR: ischemia–reperfusion
Myd88: myeloid differentiation primary response 88
*Myd88*KO: *Myd88* knockout
NeuN: neuronal nuclei
OGD: oxygen and glucose deprivation
PBS: phosphate-buffered saline
RGC: retinal ganglion cell
RT-PCR: reverse transcription polymerase chain reaction
TLR: toll-like receptor
Trif: TIR-domain-containing adapter inducing interferon-β
*Trif*KO: *Trif* knockout
WT: wild-type

## Supporting Information

Figure S1 Immunochemistry performed on retinal sections from WT, *Trif*KO and *Myd88*KO mice demonstrated the specificity of anti-Trif and anti-Myd88 antibodies.
