# Supplementary material for: The TIR-domain-containing adapter inducing interferon-β-dependent signaling cascade plays a crucial role in ischemia–reperfusion-induced retinal injury, whereas the contribution of the myeloid differentiation primary response 88-dependent signaling cascade is not as pivotal
Source: Eur J Neurosci. 2014 Apr 23;40(3):2502–12. doi: 10.1111/ejn.12603 (PMC4122625; doi:10.1111/ejn.12603)
Supplement: Figure S1 — Immunochemistry performed on retinal sections from WT, TrifKO and Myd88KO mice demonstrated the specificity of anti-Trif and anti-Myd88 antibodies. [file ejn0040-2502-sd1.pdf]

Supplement 1

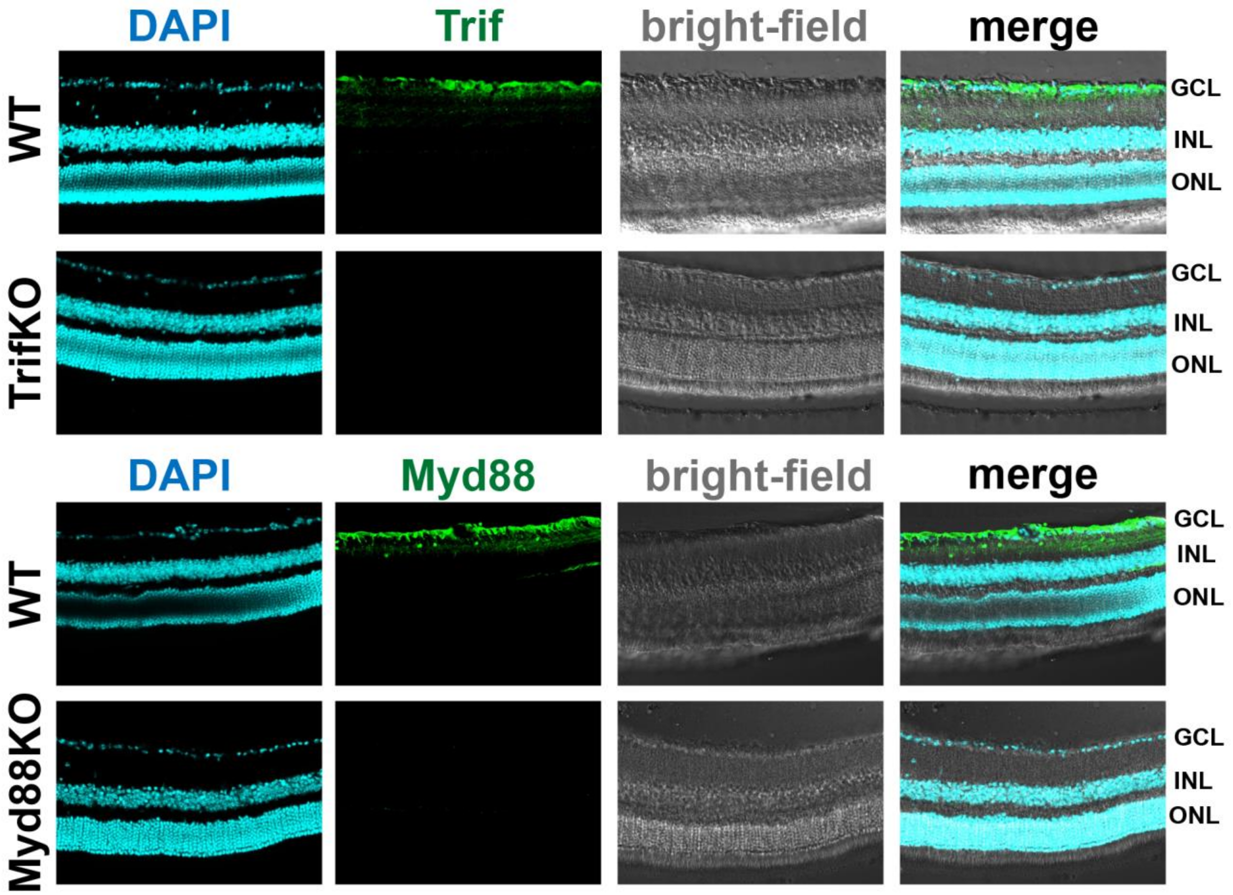

Figure S: Immunohistochemistry performed on retinal sections from wild type (WT), Trif and Myd88 knockout mice (TrifKO and Myd88KO) demonstrated specificity of anti-Trif and anti-Myd88 antibodies.
